# miR-1251-5p Overexpression Inhibits Proliferation, Migration, and Immune Escape in Clear Cell Renal Cell Carcinoma by Targeting NPTX2

**DOI:** 10.1155/2022/3058588

**Published:** 2022-03-10

**Authors:** Lin Yue, Hualong Lin, Shaofei Yuan, Lili Wu, Guangming Chen, Jiaqi Wang, Jieni Feng

**Affiliations:** ^1^School of Nursing, Hunan University of Medicine, Huaihua 418000, China; ^2^Department of Medical Oncology, The Third Affiliated Hospital of Wenzhou Medical University, Rui'an 325200, China; ^3^Department of Urology, The Third Affiliated Hospital of Wenzhou Medical University, Rui'an 325200, China

## Abstract

**Background:**

miR-1251-5p was identified as a tumor suppressor in a variety of malignancies; however, its biological function in clear cell renal cell carcinoma (ccRCC) is unknown.

**Methods:**

The Cancer Genome Atlas (TCGA) database was used to download expression information, including miR-1251-5p, in 521 ccRCC tissues and 71 ordinary tissues, and bioinformatics was used to explore possible target mRNAs. The relationship between miR-1251-5p, target mRNA activity, and clinical factors was examined. To estimate the biological activity of miR-1251-5p and target mRNA in ccRCC cells, we used MTT, colony formation, enzyme-linked immunosorbent, and Transwell assays. We employed a dual-luciferase reporter assay and a western blot to examine the molecular mechanisms of miR-1251-5p in ccRCC cells. In addition, the expressions of miR-1251-5p and target mRNA were further verified in the GEO database.

**Results:**

Our findings revealed that miR-1251-5p binds with NPTX2's 3′-UTR. In TCGA and GEO datasets, miR-1251-5p activity is found to be lower in ccRCC tissues than that in nearby conventional tissues, although NPTX2 activity is higher. In ccRCC sufferers, miR-1251-5p and NPTX2 act as biomarkers that indicate a bad prognosis. Meanwhile, in miR-1251-5p tissues, NPTX2 expression and multiple clinical variables (survival status, grade, T staging, N staging, M staging, and clinical stage) had significant differences (*p* < 0.05). Structurally, miR-1251-5p inhibited proliferation, migration, and immune escape of ccRCC cells by targeting NPTX2.

**Conclusion:**

Our findings indicate that miR-1251-5p constrained ccRCC cell advancement, migration, and immune evasion via targeting NPTX2, providing novel insights into ccRCC target treatment.

## 1. Introduction

In China, the most frequent malignant tumor of a urinary scheme is renal carcinoma, whereas the RCC is the next most common. RCC occurrence is rising in most Asian nations. For example, RCC is increasing in men in China at 7.7 percent yearly growth. Affected regions in China are Hong Kong and Shanghai. Meanwhile, women's RC prevalence has increased by 2.0% annually in India, with Chiang Mai and Mumbai being the worst affected. The National Cancer Spectrum of China shows 6.6 percent annual growth in RC prevalence in the last 20 years and 40 percent of people dying from RCC. It is estimated that 20 to 30 percent of RCC sufferers have the recurring or metastatic disease after their first trip to the hospital, and this malignancy is responsible for over 1 million fatalities each year.

The ccRCC has been the 13th commonly diagnosed cancer all over the world in 2020, which brings deep influence on human health [1]. At present, the biomarker study about ccRCC has made great progress, and microRNAs (miRNAs) are increasingly being discovered for having a function in the occupancy and growth of ccRCC [2].

miRNAs are a kind of noncoding RNAs with more than 20 nucleotides that have no potential to encode proteins [3]. Several miRNAs have been known to have a great influence on proliferation and metastasis of ccRCC cells, such as miR-5710, miR-335, and miR-155. miR-5710 binds transcription factor PDE1B, thus inactivating transcriptional activity of complexes, which inhibits apoptosis of ccRCC cells [4]. miR-335 upregulates the expression of KDM3A and YAP1, which shows carcinogenic effect in ccRCC [5]. In ccRCC lines, miR-155 suppresses FOXO3 expression, accelerating cellular tumor growth [6]. miR-1251-5p was found on chromosome 12q23.1, but there were few studies about this miRNA in ccRCC. Liang et al. [7] and Zhou et al. [8] have confirmed that miR-1251-5p is a prognostic factor of ccRCC based on database analysis, but no experimental verification has been carried out and only TCGA database has been used for analysis. The activity of miR-1251-5p is upregulated with hepatocellular carcinoma cells, according to a microarray study of miRNAs, and could represent a viable therapeutic target for therapy [9]. miR-1251-5p acts as an oncogene in ovarian cancer, suppressing TBCC and *α*/*β*-tubulin expression [10]. However, the potential molecular system of miR-1251-5p in ccRCC was never explored and not confirmed by functional experiments.

NPTX2 binds to its receptor, NPTXR, as an extracellular ligand [11]. The importance of NPTX2/NPTXR axis in nervous system disorders [12], which entails the activation of glutamate receptors as well as the formation of synapses, has been discovered via extensive research. In female mice having anxiety and depression, NPTX2 expression has been reduced [13]. NPTX2 was shown to be overexpressed in colorectal cancer tissues in this investigation, and T stages were connected to increased NPTX2 expression, lymph node invasion, distant metastases, clinical step, and bad prognosis in colorectal cancer patients [14]. von Roemeling et al. [15] revealed that NPTX2 promotes cell migration by interacting with the GluR4 subunit of the -amino-3-hydroxy-5-methyl-4-isoxazole propionic acid receptor. In addition, Xiang et al. [16] discovered a new regulatory system for miR96 regulating NPTX2 expression in ccRCC cases. The molecular basis of NPTX2 with miR-1251-5p in ccRCC, however, was never investigated.

We discovered that miR-1251-5p has been downregulated in ccRCC cell lines in this investigation. miR-1251-5p acted as a tumor inhibitor by inhibiting cell proliferation, migration, and immune evasion by targeting NPTX2.

## 2. Materials and Methods

### 2.1. Microarray and Clinical Data

TCGA database was used to obtain RNA-sequence data and clinical data from ccRCC patients, comprising 521 ccRCC tissues and 71 surrounding normal tissues. After incomplete information on clinical factors was deleted, 511 patients having ccRCC have been included in the study for further investigation.

The gene expression characteristics and clinical information (gender, age, and surviving condition) of patients with ccRCC were added to TCGA database. The dual-luciferase reporter assay and western blot methods have been employed to analyze the immune and survival values in ccRCC cases. All of the patients were separated into two groups: cancerous and healthy (Table 1).

### 2.2. Exploring Clinical Benefit

NPTX2 and miR-1251-5p for predicting survival were assessed by the area under the curve (AUC) and receiver operating characteristic (ROC) curve. To evaluate the cutoff value within the ROC curve, we computed individual patients' risk score, which was used to identify “high-risk” and “low-risk” groups. The Kaplan–Meier survival test suggests survival differences between high-risk and low-risk groups.

### 2.3. Cell Culture

Human ccRCC cell lines 786-O, A498, ACHIN, and Caki-2 and also human ordinary epithelial cell line HK-2 were supplied by the Chinese Academy of Sciences in Shanghai. Every one of the cells being cultured in DMEM media contained a lot of glucose (Gibco, USA). Penicillin (100 U/ml) and streptomycin (100 g/ml) had been mixed with 10% fetal bovine serum (PS; Servicebio, China) (FBS; Gibco, USA). 0.25% trypsin-EDTA solution (Servicebio, China) was employed for cell passage cultivation. The cells were cultured in a cell culture incubator at 37°C and 5% CO_2_.

### 2.4. Cell Transfection

Lipofectamine®2000 was employed to transmit miR-1251-5p mimic-NC, sh-NC, sh-NPTX2, oe-NC, and oe-NPTX2 into A498 as well as Caki-2 (Invitrogen, USA). GENEray created all of the combinations (Shanghai, China).

### 2.5. RNA Extraction and Reverse Transcription

Overall RNA was extracted from 786-O, A498, ACHIN, Caki-2, and HK-2 cells utilizing TRIzol reagent (Invitrogen, USA), and 1000 ng overall RNA was utilized in first-strand cDNA using by Hifair® III 1st Strand cDNA Synthesis SuperMix for qPCR (YEASEN, China) and miRNA 1st Strand cDNA Synthesis Kit (Vazyme, China).

### 2.6. Real-Time PCR

During actual PCR, Hieff UNICON® qPCR SYBR Green Master Mix was utilized (YEASEN, China). NPTX2 expression is normalized with GAPDH, whereas miR-1251-5p expression is normalized with U6. All of the primers were created by GENEray (Shanghai, China). The 2^−ΔΔCt^ technique was employed for determining the levels of expression.

### 2.7. MTT Assay and Colony Formation Assay

For the MTT test, transfected A498 and Caki-2 cells were served in 96-well plates at the density of 1000 cells per well; thereafter, the growth of the cell was estimated at 24 h, 48 h, 72 h, 96 h, and 120 h. The supernatants were collected after 6 h of observation at 37°C using 5% CO_2_, and 150l DMSO was injected into every well to disintegrate formazan (Sangon, China), with the absorbance reading recorded at 490 nm. Again for colony formation assay, transfected A498 and Caki-2 cells were served in 6-well plates at a density of 500 cells per well, and cells were observed for 7–14 days at 37°C using 5% CO_2_ and refreshed per 3 days. The medium was replaced after the clones were detectable, and the cells were rinsed 3 times using phosphate buffer saline (PBS, Servicebio, China), preserved for 10 min with 95% ethanol, and stained with 0.1% crystal violet (YEASEN, China). Relevant photographs were taken using the camera.

### 2.8. Western Blot Analysis

A BCA Protein Quantification Kit was used to assess protein content and RIPA lysis solution (YEASEN, China) (YEASEN, China). For polyacrylamide gel electrophoresis (PAGE), 30 g total protein was used and nitrocellulose membranes were used to transmit the proteins identified inside the gel (Pall, USA). Upon the transfer, the membrane was incubated using 5% nonfat milk for 1 hour until being incubated with primary antibodies to NPTX2 for 12–18 hours at 4°C (CST, USA) and GAPDH (Abclonal, China). Then, membranes were rinsed 3 times using Tris-buffered saline Tween and blocked with secondary antibody IRDye® 800CW goat anti-rabbit IgG for one hour at room temperature (LICOR, USA).

### 2.9. Dual-Luciferase Reporter Assay

The expression of firefly luciferase and Renilla luciferase was identified with dual-luciferase reporter gene assay kit methodology (Promega, USA).

### 2.10. Enzyme-Linked Immunosorbent Assay (ELISA)

Nanjing Biotech provided activated peripheral blood mononuclear cells (PBMCs). Using phytohaemagglutinin, generated PBMCs were cocultured with A498 and Caki-2 cells over 24 hours in a humidified incubator (PHA; Invitrogen). The concentration of interferon (IFN-*α*) and tumor necrosis factor (TNF-*γ*) were determined employing IFN-ELISA kit as well as the TNF-ELISA kit (Invitrogen) as directed by the manufacturer.

### 2.11. Statistical Analysis

The information investigation was done using GraphPad Prism, which was given as mean ± standard deviation (SD). Regarding two-group comparisons, Student's *t*-test is applied. *p* < 0.05 is viewed as a crucial difference. All of the trials were done in triplicate.

## 3. Results

### 3.1. Expression, Survival Prediction, and Correlation of Clinical Parameters of miR-1251-5p in ccRCC Patients

**Figure 1:**
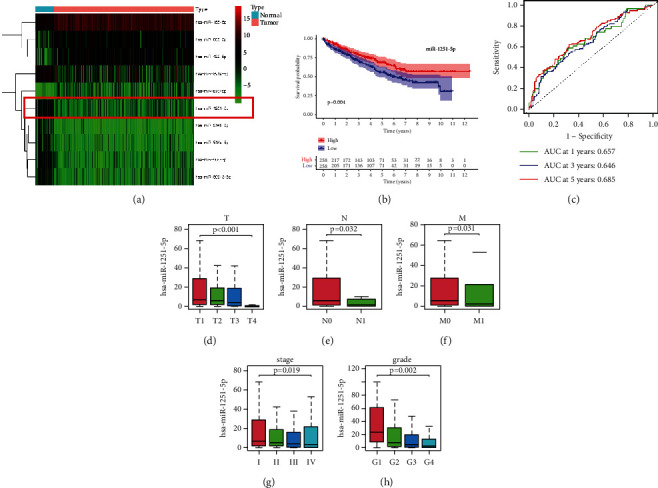
Expression, survival prediction, and correlation of clinical parameters of miR-1251-5p in ccRCC patients. (a) Heatmap for miRNAs shows the darker the color, the greater the level of expression. (b) High-expression groups have higher life durations over low-expression groups, corresponding to a Kaplan–Meier survival study. The high-expression group is depicted by the red line, while the low-expression group is depicted by the blue line. (c) Risk signature-based time-dependent ROC lines indicate one-year, three-year, and five-year survival. (d) Interaction of the miR-1251-5p using T staging. (e) Interaction of the miR-1251-5p using N staging. (f) Interaction of the miR-1251-5p using M staging. (g) Interaction of the miR-1251-5p using clinical stage. (h) Interaction of the miR-1251-5p using grade. ^*∗*^*p* < 0.05 and ^*∗∗*^*p* < 0.01.

To examine the expression of miR-1251-5p in breast cancer, we downloaded microarray data including 521 ccRCC tissues and 71 adjacent ordinary tissues from TCGA database and computed the expression of miR-1251-5p. As shown in our results, in most ccRCC tissues, expression of miR-1251-5p is minimum compared to ordinary tissues (Figures 1(a) and S1B). Meanwhile, Kaplan–Meier survival findings indicate the groupings with low expression possess short survival periods than those with high risk (*p*=0.004), as shown in Figure 1(b). Furthermore, the area under the ROC curve research stated the miR-1251-5p might be used to forecast the prognosis of ccRCC patients (0.657 at 1 year, 0.646 at 3 years, and 0.685 at 5 years), as shown in Figure 1(c). We examine the relationship between miR-1251-5p expression and clinical factors in ccRCC patients to see if it had any clinical benefit, including T staging, N staging, M staging, grade, and clinical stage. Especially, miR-1251-5p expression is considerably greater in T1 cases (Figure 1(d)), N0 cases (Figure 1(e)), M0 cases (Figure 1(f)), early-stage cases (Figure 1(g)), and early-grade cases (Figure 1(h)).

Our outcomes depict such an amount of miR-1251-5p expression is linked to survival prediction as well as clinical factors.

### 3.2. Expression, Survival Prediction, and Correlation of Clinical Parameters of NPTX2 in ccRCC Patients

**Figure 2:**
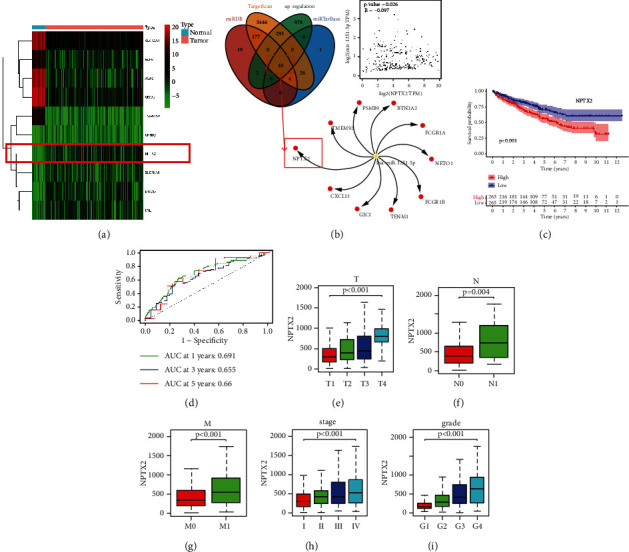
Expression, survival prediction, and correlation of clinical parameters of NPTX2 in ccRCC patients. (a) Heatmap for mRNAs shows that the darker the color, the greater the level of expression. (b) The intersection of miRDB database, StarBase database, TargetScan database, and upregulation of mRNAs for potential regulatory factors. (c) High-expression groups have higher survival time than low-expression groups, corresponding to a Kaplan–Meier survival study. The high-expression group is expressed by the red line, while the low-expression group is expressed by the blue line. (d) Risk signature-based time-dependent ROC lines indicating 1-year, three-year, and five-year survival. (e) Correlation of the NPTX2 with T staging. (f) Correlation of the NPTX2 with N staging. (g) Correlation of the NPTX2 with M staging. (h) Correlation of the NPTX2 with clinical stage. (i) Correlation of the NPTX2 with grade. ^*∗*^*p* < 0.05 and ^*∗∗*^*p* < 0.01.

**Figure 3:**
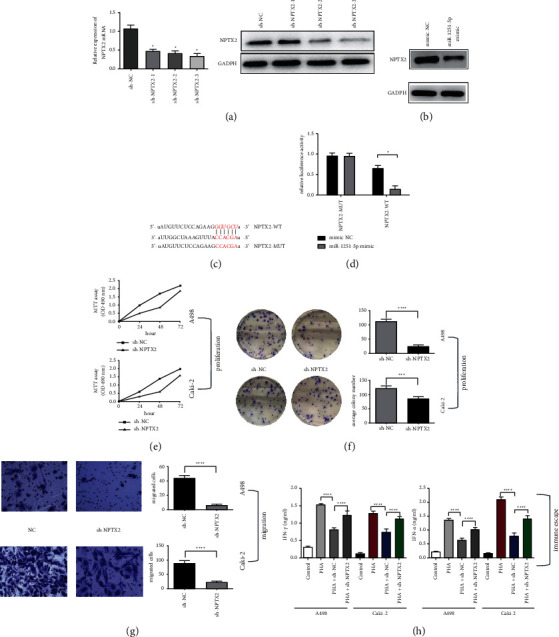
Loss function of NPTX2 inhibits proliferation, migration, and immune escape of ccRCC cells. (a) mRNA and protein expression level in NPTX2-silenced ccRCC cell line. (b) Overexpression of miR-1251-5p suppressed expression of NPTX2 in protein level. (c) The association between miR-1251-5p and mutant category NPTX2 3′-UTR is depicted. (d) In cell lines cotransfected using wild/mutant type NPTX2 3′-UTR and miR-1251-5p copies, dual-luciferase tests were performed. (e) ccRCC cells of A498 and Caki-2 cells after knockdown of NPTX2 at 24 hours, 48 hours, and 72 hours are computed using MTT assay, respectively. (f) After knocking down NPTX2, the “colony formation assay” has been executed to compute cell's capability in A498 and Caki-2 cells. (g) “Transwell assay” is used to explore the potential of A498 and Caki-2 cells to migrate after knockdown of NPTX2 (100×). (h) An ELISA kit was used to assess the levels of IFN-*α* and TNF-*γ* in the supernatants of A498 and Caki-2 cells. ^*∗*^*p* < 0.05 and ^*∗∗*^*p* < 0.01.

To locate the objective gene of miR-1251-5p, we analyzed TCGA dataset and screened the expression of 1283 genes that were negatively tested with the expression of miR-1251-5p in ccRCC. Meanwhile, we searched the miRDB database, StarBase database, and TargetScan database for potential regulatory factors. Finally, we identified 10 genes from the intersection as potential targets of miR-1251-5p, including NPTX2, GJC1, BTN3A2, TENM1, TMEM92, PSMB9, FCGR1B, CXCL11, FCGR1A, and NETO1 (Figure 2(b)). Among the 10 genes, only NPTX2 expression was most significantly associated with survival status (*p* < 0.001, as shown in Figure 2(c)). As a result, we concentrated on NPTX2 with molecular mechanisms of miR-1251-5p . Using a luciferase reporter assay, we verified the association of miR-1251-5p with NPTX2 in the following part (Figure 3(c)). In ccRCC tissues, NPTX2 expression should be greater than in healthy tissues (Figures 2(a) and S1A). NPTX2 is negatively tested with the expression of miR-1251-5p, as shown in Figure 2(b). As with miR-1251-5p, the area under the ROC curve analysis indicated that NPTX2 could predict OS in ccRCC patients (0.691 at 1 year, 0.655 at 3 years, and 0.680 at 5 years), as depicted in Figure 2(d). Besides, NPTX2 expression is significantly greater in T4 cases (Figure 2(e)), N1 cases (Figure 2(f)), M1 cases (Figure 2(g)), advanced-stage cases (Figure 2(h)), and advanced-grade cases (Figure 2(i)).

Our outcomes depict such NPTX2 is a possible target gene for miR-1251-5p and also NPTX2 expression levels are directly related to clinical features as well as survival conditions.

### 3.3. Overexpression of miR-1251-5p Inhibits Proliferation, Migration, and Immune Escape of ccRCC Cells

**Figure 4:**
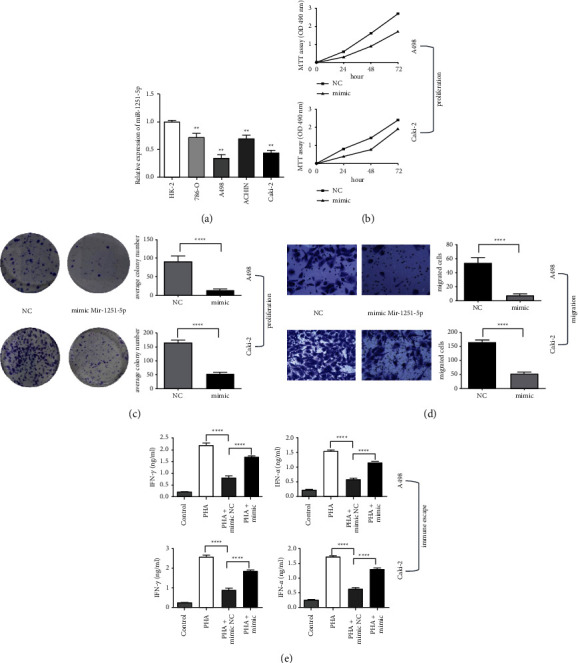
Gain function of miR-1251-5p inhibits proliferation, migration, and immune escape of ccRCC cells. (a) The expression of miR-1251-5p in cell lines is identified using qRT-PCR. (b) MTT assay is used to determine cell's activity in A498 and Caki-2 cell lines after 24 h, 48 h, and 72 h. (c) The proliferation of A498 and Caki-2 cells is identified applying the colony formation test. (d) Transwell has been utilized to test the capability of A498 and Caki-2 cell lines to migrate. (e) An ELISA kit is used to assess the levels of IFN-*α* and TNF-*γ* in the supernatant of A498 and Caki-2 cells. ^*∗*^*p* < 0.05 and ^*∗∗*^*p* < 0.01.

In a normal cell line (HK-2) and four cancer cell lines, we found expression of miR-1251-5p (786-O, A498, ACHIN, and Caki-2); similarly, expression of miR-1251-5p is minimum in cancer cell lines compared to ordinary epithelial cell line (Figure 4(a)). We also transfected A498 and Caki-2 cells using a miR-1251-5p mimic to achieve the gain function of miR-1251-5p, and the effectiveness of overexpression is determined by real-time PCR. Proliferation (Figures 4(b) and 4(c)) and migration (Figure 4(d)) of ccRCC cells were inhibited after the overexpression of miR-1251-5p. Furthermore, coculturing with mimic-NC-transfected cells decreased cell supernatant levels of IFN-*α* and TNF-*γ* upregulated in PHA-stimulated PBMC, but coculturing with mimic-transfected cells blocked this immune-suppressive action (Figure 4(e)).

Our outcomes imply that miR-1251-5p inhibited ccRCC cell growth, migration, and immune evasion in vitro.

### 3.4. Knockdown of NPTX2 Inhibits Proliferation, Migration, and Immune Escape of ccRCC Cells

**Figure 5:**
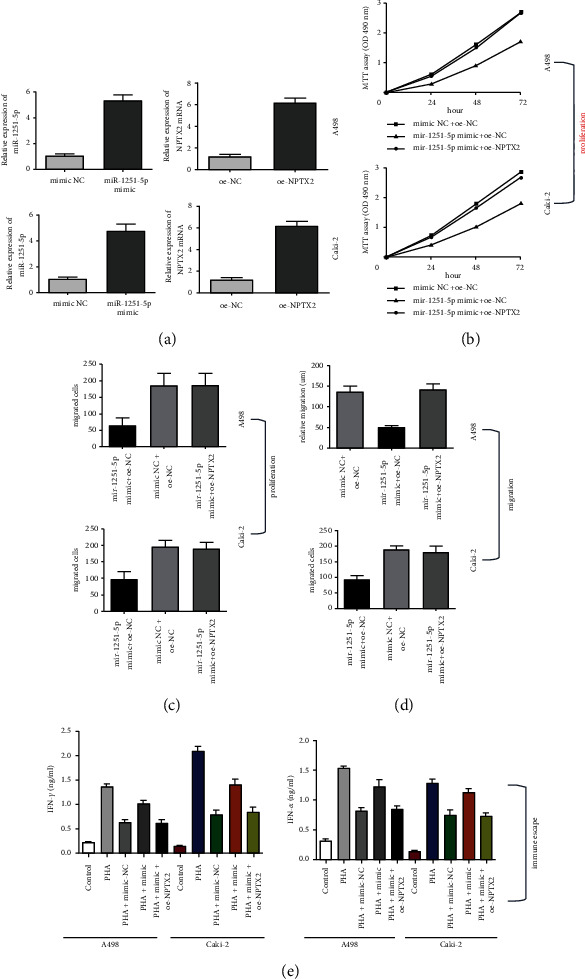
NPTX2 can reverse the effects of miR-1251-5p overexpression. (a) The expression of miR-1251-5p and NPTX2 in transfected cells are identified using qRT-PCR. (b) A498 and Caki-2 cells after transfected with oe-NPTX2 and mimic-mir-1251-5p at 24 hours, 48 hours, and 72 hours are evaluated using MTT assay, correspondingly. (c) The viability of A498 and Caki-2 cells upon transaction with oe-NPTX2 and mimic-mir-1251-5p is determined using the colony formation assay. (d) The capacity of A498 and Caki-2 cells to migrate after being transfected with oe-NPTX2 and mimic-mir-1251-5p is tested using Transwell assay. (e) The levels of IFN-*α* and TNF-*γ* in the A498 and Caki-2 cell supernatant are estimated with an ELISA kit. ^*∗*^*p* < 0.05 and ^*∗∗*^*p* < 0.01.

The NPTX2 expression level was downregulated dramatically in ccRCC cell lines transfected with three sh-NPTX2 (*p* < 0.01), as shown in Figure 3(a). We carried out further studies using sh-NPTX2-3 because it was able to significantly downregulate NPTX2 expression in A498 and Caki-2. To verify the association between miR-1251-5p and NPTX2, we used a luciferase reporter experiment with a pmirGLO-NPTX2 3′-UTR-wild vector as well as a pmirGLO-NPTX2 3′-UTR-mutant vector. Our findings revealed that miR-1251-5p can bind to the 3′-UTR of NPTX2 mRNA, increasing its degradation (Figures 3(c) and 3(d)). Furthermore, we used western blot to identify changes in NPTX2 protein expression, and the findings revealed that miR-629-5p may suppress NPTX2 expression (Figure 5(b)). Finally, we marked the biological function of NPTX2 in ccRCC, as our findings showed shRNA-induced knockdown of NPTX2 constrained proliferation, migration, and immune escape of ccRCC cells (Figures 5(e)), which indicated that NPTX2 was a tumor driver in ccRCC.

Taken together, our results revealed that NPTX2 could combine with miR-1251-5p and downregulation of NPTX2 constrains proliferation, migration, and immune escape of ccRCC in vitro.

### 3.5. NPTX2 Can Reverse the Effects of miR-1251-5p Overexpression

We investigated the effect of mimic-miR-1251-5p and oe-NPTX2 on ccRCC based on the previously mentioned negative correlation between miR-1251-5p and NPTX2. Firstly, the effectiveness of miR-1251-5p and NPTX2 overexpression was investigated (Figure 5(a)). The consequences of miR-1251-5p on A498 and Caki-2 cells might be mitigated by NPTX2 overexpression, according to the findings of MTT, colony formation, Transwell, and enzyme-linked immunosorbent assays (Figures 5(b)–5(e)).

Our findings show the overexpression of miR-1251-5p suppresses ccRCC cell proliferation, motility, and immune escape that can be mitigated by NPTX2.

## 4. Discussion

miRNAs play a major role in the development and advancement of several malignancies, renal cell carcinoma, and kidney disease. The function of miR-1251-5p is the only establishment. In ovarian cancer cells, by addressing the tumor suppressor TBCC, Zhou et al. [8] found that miR-1251-5p increased carcinogenesis and autophagy. However, it is unknown if miR-1251-5p performs a function in developing HCC [7]. Several functional assays were carried out in this work, and the findings revealed that miR-1251-5p suppressed ccRCC cell proliferation, migration, and immune escape in vitro. Our outcomes imply that miR-1251-5p operations as an antioncogene in ccRCC. In this work, there was enough evidence to conclude that NPTX2 is a specific functional goal of miR-1251-5p in ccRCC. In ccRCC tissues, miR-1251-5p was shown to be negatively linked with NPTX2 mRNA levels. Second, miR-1251-5p had a substantial impact on the luciferase expression of vectors containing the wt-3′-UTR of NPTX2, but not the mut-3′-UTR. Finally, miR-1251-5p relaxed the expression of NPTX2 in ccRCC cells on the mRNA and protein levels. Critically, restoring NPTX2 expression in ccRCC cells may be able to reverse the action of miR-1251-5p.

C-Met, NPTX2, NEUROD6, and hyperpolarization-activated cyclic nucleotide-gated potassium channel 1 have all been found to be HCN1 in a study comparing gene expression in Alzheimer's and nondisordered Alzheimer's diseases [12]. Furthermore, we discovered that NPTX2 expression in ccRCC was greater than in the normal tissue. NPTX2 was shown to improve cell migration and proliferation, according to our findings. These findings demonstrated that NPTX2-expressing cells had more ability to migrate to the ccRCC, ensuring increased NPTX2 expression in ccRCC tissues.

In conclusion, our findings highlighted the bioactivity and molecular mechanism of miR-1251-5p in ccRCC, revealing new information about ccRCC target treatment.

## 5. Conclusions

The bioactivity of miR-1251-5p in ccRCC was recognized in the research. TCGA search was performed for miR-1251-5p data including target mRNAs. Our study looked at miR-1251-5p, target mRNA expression, and clinical factors. The bioactivity of miR-1251-5p and target mRNA has been estimated in ccRCC cells using MTT, colony formation, enzyme-linked immunosorbent, and Transwell assays. This miR-1251-5p was also studied utilizing the dual-luciferase reporter assay with western blot. The GEO data were used to confirm miR-1251-5p with target mRNA activity. The 3′-UTR of NPTX2 binds miR-1251-5p. In TCGA and GEO databases, miR-1251-5p activity was found less in ccRCC versus normal tissues. As a result of our findings, we now know that miR-1251-5p can reduce ccRCC cell proliferation, migration, and immune evasion via target NPTX2.

## Figures and Tables

**Table 1:** Clinical information with variables and their respective count of two groups: cancerous and healthy.

Variables	Count (*n* = 592)
Age	
Less than or equal to 50	281
Greater than 50	311
Gender	
Male	364
Female	222
NA	6
Tissues	
Cancerous	521
Normal	71

## Data Availability

The data used to support the findings of the study were obtained from TCGA database and the Kaplan–Meier Plotter database.

## Supplementary Materials

Supplementary Materials Figure S1: NPTX2 and miR-1251-5p expression in GEO datasets. (A) miR-1251-5p expression in GEO datasets; (B) NPTX2 expression in GEO datasets.

## References

[1] Akhtar M., Al-Bozom I. A., Al Hussain T. (2019). Papillary renal cell carcinoma (PRCC): an update. *Advances in Anatomic Pathology*.

[2] Cheng L., Cao H., Xu J. (2021). Circ_RPL23A acts as a miR-1233 sponge to suppress the progression of clear cell renal cell carcinoma by promoting ACAT2. *Journal of Bioenergetics and Biomembranes*.

[3] Bushati N., Cohen S. M. (2007). microRNA functions. *Annual Review of Cell and Developmental Biology*.

[4] Zhao C., Mo L., Lei T. (2021). miR-5701 promoted apoptosis of clear cell renal cell carcinoma cells by targeting phosphodiesterase-1B. *Anti-Cancer Drugs*.

[5] Zhang W., Liu R., Zhang L. (2021). Downregulation of miR-335 exhibited an oncogenic effect via promoting KDM3A/YAP1 networks in clear cell renal cell carcinoma. *Cancer Gene Therapy*.

[6] Meng L., Xing Z., Guo Z., Qiu Y., Liu Z. (2021). Hypoxia-induced microRNA-155 overexpression in extracellular vesicles promotes renal cell carcinoma progression by targeting FOXO3. *Aging*.

[7] Liang B., Zhao J., Wang X. (2017). A three-microRNA signature as a diagnostic and prognostic marker in clear cell renal cancer: an in Silico Analysis. *PLoS One*.

[8] Zhou Q., Zhang Z.-Y., Ang X.-J., Hu C., Ouyang J. (2021). Construction of five microRNAs prognostic markers and a prognostic model for clear cell renal cell carcinoma. *Translational Cancer Research*.

[9] Han S., Wang L., Sun L. (2020). MicroRNA-1251-5p promotes tumor growth and metastasis of hepatocellular carcinoma by targeting AKAP12. *Biomedicine & Pharmacotherapy*.

[10] Shao Y., Liu X., Meng J., Zhang X., Ma Z., Yang G. (2019). MicroRNA-1251-5p promotes carcinogenesis and autophagy via targeting the tumor suppressor TBCC in ovarian cancer cells. *Molecular Therapy*.

[11] Chang S., Bok P., Tsai C.-Y. (2018). NPTX2 is a key component in the regulation of anxiety. *Neuropsychopharmacology*.

[12] Xiao M.-F., Xu D., Craig M. T. (2017). NPTX2 and cognitive dysfunction in Alzheimer’s Disease. *Elife*.

[13] Belbin O., Xiao M.-F., Xu D. (2020). Cerebrospinal fluid profile of NPTX2 supports role of Alzheimer’s disease-related inhibitory circuit dysfunction in adults with Down syndrome. *Molecular Neurodegeneration*.

[14] Xu C., Tian G., Jiang C. (2019). NPTX2 promotes colorectal cancer growth and liver metastasis by the activation of the canonical Wnt/*β*-catenin pathway via FZD6. *Cell Death & Disease*.

[15] von Roemeling C. A., Radisky D. C., Marlow L. A. (2014). Neuronal pentraxin 2 supports clear cell renal cell carcinoma by activating the AMPA-selective glutamate receptor-4. *Cancer Research*.

[16] Xiang W., Han L., Mo G. (2020). MicroRNA‐96 is a potential tumor repressor by inhibiting NPTX2 in renal cell carcinoma. *Journal of Cellular Biochemistry*.

