# Supplementary material for: miR-1251-5p Overexpression Inhibits Proliferation, Migration, and Immune Escape in Clear Cell Renal Cell Carcinoma by Targeting NPTX2
Source: J Oncol. 2022 Mar 10;2022:3058588. doi: 10.1155/2022/3058588 (PMC8930236; doi:10.1155/2022/3058588)
Supplement: Supplementary Materials — Figure S1: NPTX2 and miR-1251-5p expression in GEO datasets. (A) miR-1251-5p expression in GEO datasets; (B) NPTX2 expression in GEO datasets. [file 3058588.f1.docx]

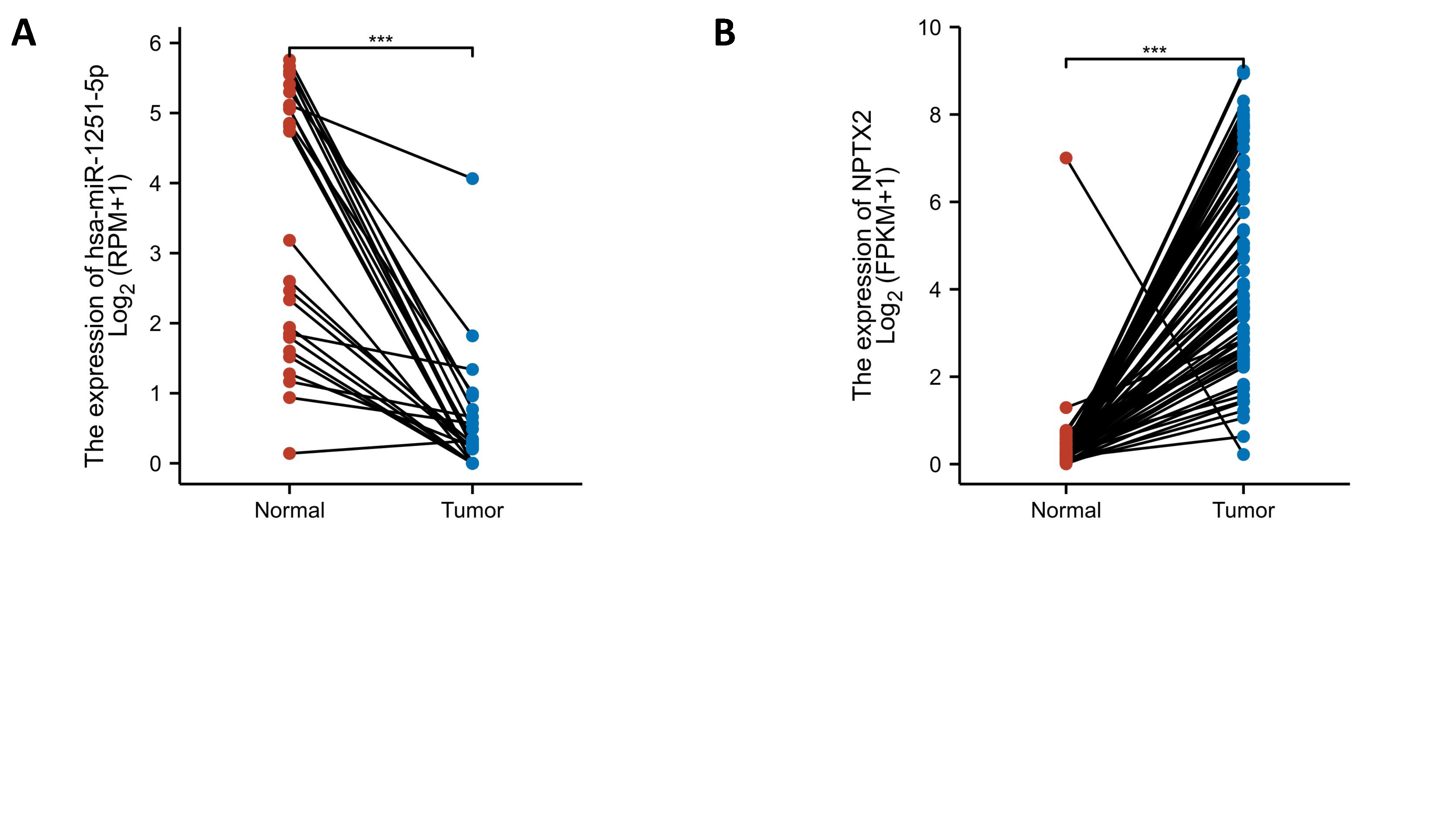


**Fig.S1 *NPTX2* and miR-1251-5p expression in GEO datasets;** miR-1251-5p expression in GEO datasets**; (B)** *NPTX2* expression in GEO datasets
